# Mutations in BRCA1, BRCA2 and other breast and ovarian cancer susceptibility genes in Central and South American populations

**DOI:** 10.1186/s40659-017-0139-2

**Published:** 2017-10-06

**Authors:** Lilian Jara, Sebastian Morales, Tomas de Mayo, Patricio Gonzalez-Hormazabal, Valentina Carrasco, Raul Godoy

**Affiliations:** 1 Human Genetics Program, Institute of Biomedical Sciences (ICBM), School of Medicine, University of Chile, Santiago, Chile; 2 Center for Genetics and Genomics Faculty of Medicine, Clinica Alemana Universidad del desarrollo, Avenida Las Condes, 12438 Lo Barnechea, Santiago, Chile; 3 Laboratorio de Genética Molecular Humana, Facultad de Medicina, Instituto de Ciencias Biomédicas (ICBM), Programa de Genética, Universidad de Chile, Independencia 1027, Santiago, Chile

**Keywords:** Hereditary and early onset breast cancer, Susceptibility genes, Pathogenic point mutations, Large genomic rearrangements, Ethnic composition

## Abstract

Breast cancer (BC) is the most common malignancy among women worldwide. A major advance in the understanding of the genetic etiology of BC was the discovery of *BRCA1* and *BRCA2* (*BRCA1/2*) genes, which are considered high-penetrance BC genes. In non-carriers of *BRCA1/2* mutations, disease susceptibility may be explained of a small number of mutations in *BRCA1/2* and a much higher proportion of mutations in ethnicity-specific moderate- and/or low-penetrance genes. In Central and South American populations, studied have focused on analyzing the distribution and prevalence of *BRCA1/2* mutations and other susceptibility genes that are scarce in Latin America as compared to North America, Europe, Australia, and Israel. Thus, the aim of this review is to present the current state of knowledge regarding pathogenic *BRCA* variants and other BC susceptibility genes. We conducted a comprehensive review of 47 studies from 12 countries in Central and South America published between 2002 and 2017 reporting the prevalence and/or spectrum of mutations and pathogenic variants in *BRCA1/2* and other BC susceptibility genes. The studies on *BRCA1/2* mutations screened a total of 5956 individuals, and studies on susceptibility genes analyzed a combined sample size of 11,578 individuals. To date, a total of 190 different *BRCA1/2* pathogenic mutations in Central and South American populations have been reported in the literature. Pathogenic mutations or variants that increase BC risk have been reported in the following genes or genomic regions: *ATM*, *BARD1, CHECK2, FGFR2, GSTM1, MAP3K1, MTHFR, PALB2, RAD51, TOX3, TP53, XRCC1*, and *2q35*.

**Electronic supplementary material:**

The online version of this article (doi:10.1186/s40659-017-0139-2) contains supplementary material, which is available to authorized users.

## Background

Breast cancer (BC) is the most common malignancy among women worldwide. Each year, 1.15 million new cases are diagnosed, representing 23% of all cancer diagnoses among women [1, 2], and one in eight women will develop BC during their lives [3]. The greatest challenge currently facing clinical researchers, therefore, is identifying prevention strategies that would reduce the morbidity and mortality associated with the disease.

Breast cancer (BC) is a complex disease, with both sporadic and familial presentations, as in most cancers. Inherited genetic risk factors contribute to BC susceptibility in both familial and sporadic BC.

The discovery of tumor suppressor genes BRCA1 (MIM 113705) and BRCA2 (MIM 600185) [4, 5] was a major advance in elucidating the genetic etiology of BC. A mutation that inactivates the BRCA proteins increases the risk for breast, ovarian, and other cancers. These genes are now considered high-penetrance dominant autosomal genes for BC susceptibility. Germline mutations in *BRCA1* and *BRCA2* are responsible for about 25% of the risk for familial BC [6–8] and therefore 5–10% of all BC cases [9]. Retrospective studies [10–19], suggest an estimated cumulative risk of breast cancer to 70 years of age of 40–87% for *BRCA1* carriers and 27–84% for *BRCA2* carriers. The corresponding ovarian cancer risks are 16–68% for *BRCA1* carriers and 11–30% for *BRCA2* carriers. Disease-causing mutations are distributed throughout the entire coding regions of both genes. Since the identification of *BRCA1/2* as the principal genes responsible for inherited BC [5, 20], over 3781 distinct DNA sequence variants have been added to the BIC database (http://research.nhgri.nih.gov/bic/). Of these, 3079 are classified as pathogenic, including 1598 truncating mutations (1197 frameshift and 387 nonsense) and 14 splicing alterations. The frequency of BRCA1/2 mutations varies significantly according to geographic region and ethnicity.

There is a consensus that mutations in genes BRCA1/2 and TP53 are responsible for on average 16–20% of the risk for familial BC [6, 7]. Genome-wide linkage analyses using large samples of BRCA1/2-negative families have not mapped any other high-penetrance susceptibility loci to date [21]. Therefore, a large part of the genetic component remains unidentified. How can the remaining ~ 80% of familial BC risk be explained? Ford et al. [15] proposed that other susceptibility alleles, called moderate- or low-penetrance, could be responsible for a significant percentage of BC in BRCA1/2-negative families. Currently, BC risk variants can be classified into three categories of penetrance (high, moderate, and low) that reflect the probability of developing the disease [22]. Therefore, in non-carriers of BRCA1/2 mutations, disease susceptibility may be explained by mutations in other high-, moderate- or low-penetrance genes, interactions between alleles involved in the same pathways, or environmental factors. Sporadic BC is the result of serial stepwise accumulation of acquired and uncorrected mutations in somatic genes that are yet to be identified [23]. Nevertheless, in cases without a family history of BC (sporadic BC), certain combinations of low-penetrance alleles that are associated with a high polygenic risk score (PRS) have been shown to contribute to BC susceptibility [22].

Screening for BRCA1 and BRCA2 mutations provides potentially significant health benefits. Armed with genetic results, physicians may offer risk-reducing options for mutation carriers who have, thus far, not developed cancer, such as prophylactic mastectomy and oophorectomy, prophylactic tamoxifen, or surveillance [24–28].

Research evaluating the distribution and prevalence of BRCA1/2 mutations in Central and South American populations has been quite limited as compared to the number of studies in North America, Europe, Australia and Israel. Moreover, some of the studies performed in Latin America have analyzed hereditary BC, while others have evaluated early-onset BC or cohorts unselected for family history. Furthermore, because Central and South American populations are of mixed ethnic origin, the distributions of recurrent mutations vary by region and country. Published data regarding other BC susceptibility genes is even scarcer than data on BRCA1/2 mutations. Therefore, the aim of this review is to provide a report on the current state of knowledge regarding pathogenic point mutations and large genomic rearrangements (LGRs) in BRCA1 and BRCA2, as well as mutations in other BC susceptibility genes, in Central and South American populations.

## Methods

PubMed, EBSCO, and SciELO databases were searched for all studies involving BRCA1 and BRCA2 mutations in Central and South American individuals with breast cancer. Moreover, we searched for pathogenic mutations or variants in other susceptibility genes in the same populations. The search terms included “hereditary breast cancer;” “South America,” “Latin America,” and other terms associated with Central or South American countries; and “BRCA1 and BRCA2″ and “genes and breast cancer risk.” Manuscripts published through February 28, 2017 were considered. Only papers published in English or Spanish were reviewed. Non-human studies, in vitro or in vivo studies, and studies focused on topics other than breast/ovarian cancer were excluded.

The inclusion criteria varied significantly among the selected studies; therefore, we classified the articles into three categories: cohorts that included cases with hereditary BC (cohort A), cases with early-onset (≤ 40 years) BC (cohort B), and cases unselected for family history of BC (cohort C). We classified a cohort as hereditary BC (cohort A) if the inclusion criteria met one or more of the following criteria, as established in the literature: (1) At least two first-degree relatives with BC and/or ovarian cancer diagnosed at any age; (2) at least two first- or second-degree relatives with BC diagnosed before the age of 50 years; (3) at least three first- or second-degree relatives with BC with at least one diagnosed before the age of 40; (4) at least one relative with BC diagnosed before the age of 50 and at least one relative with ovarian cancer diagnosed at any age; (5) at least one male relative with BC diagnosed at any age and at least one female relative diagnosed with BC at any age; (6) at least one relative diagnosed with BC before the age of 30 and one other first- or second-degree relative diagnosed with BC at any age; and (7) at least one relative with bilateral BC and one other first- or second-degree relative with BC. A cohort was classified as early-onset BC (cohort B) if the cohort was made up entirely of BC patients diagnosed at or before 40 years of age. We classified a cohort as unselected for family history (cohort C) if none of the criteria for hereditary BC were applied in the case selection.

Pathogenic mutations are base substitutions, deletions, or duplications that inactivate the BRCA proteins. “Recurrent” refers to mutations present in several cases in at least one cohort.

### The scope of BRCA1 and BRCA2 mutations in Central and South American countries

**Fig. 1:**
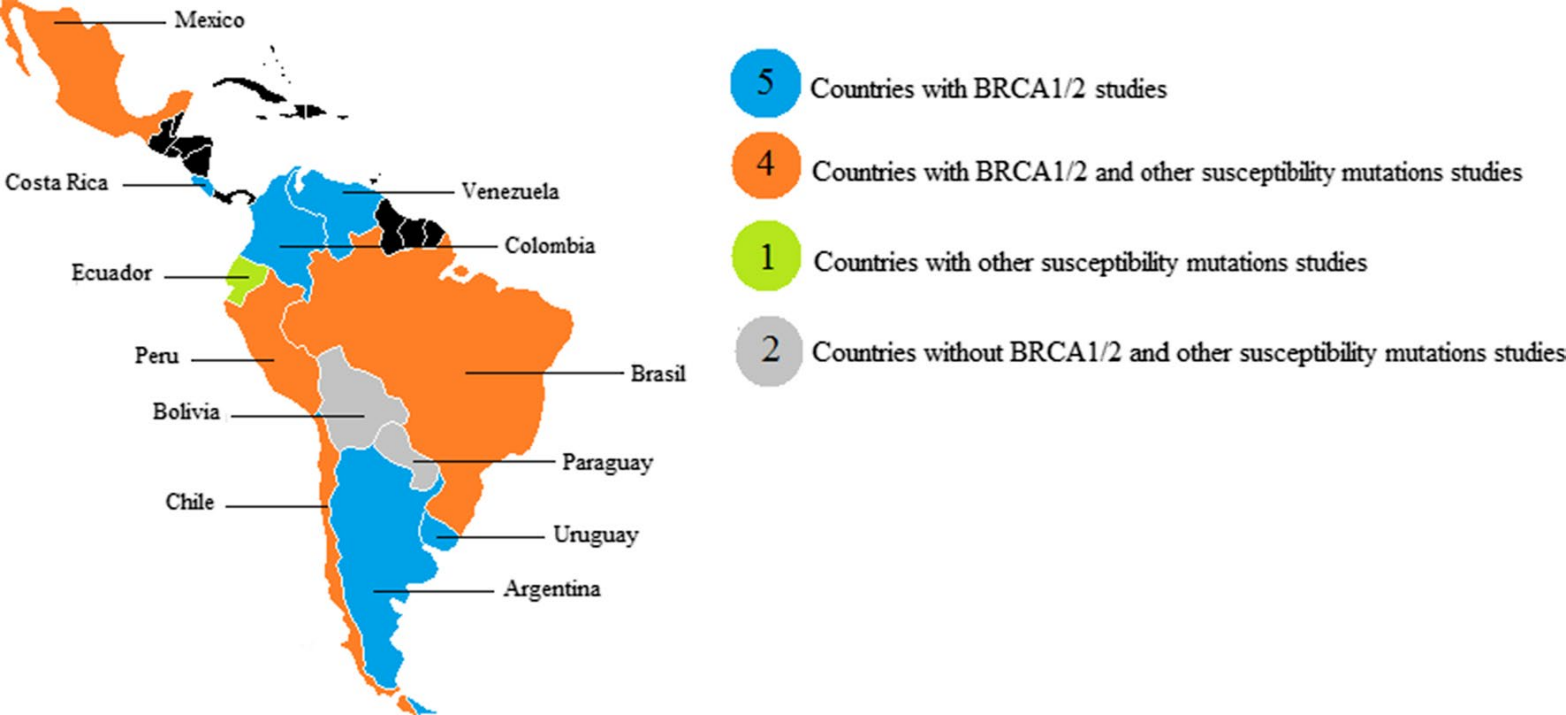
The scope of *BRCA1* and *BRCA2* mutations in Central and South American countries. In total 12 countries were evaluated. No *BRCA* mutation studies were found in Bolivia, Paraguay and Ecuador (the latter only with other susceptibility alleles)

We conducted a literature review of reports on *BRCA1* and *BRCA2* pathogenic point mutations and LGRs in 12 Central and South American countries (Argentina, Bolivia, Brazil, Chile, Colombia, Costa Rica, Ecuador, Mexico, Paraguay, Peru, Uruguay and Venezuela). Between January 2002 and February 2017, there were 28 published reports on *BRCA* mutations in these countries. Figure 1 shows that studies were performed in nine countries: Argentina, Brazil, Colombia, Costa Rica, Chile, Mexico, Peru, Uruguay and Venezuela. There were no reports on *BRCA* mutations in Bolivia, Ecuador or Paraguay. Collectively, the 28 studies screened 5956 individuals and identified 190 different pathogenic mutations (Additional file 1: Table S1; Tables 1, 2).

**Table 1:** Cohort characteristics and pathogenic BRAC1 and BRAC2 mutations in early-onset breast cancer in Central and South American populations

Country	Cohort size	Inclusion criteria	Number of mutations detected		Pathogenic mutation in BC patients				Recurrent mutation (frequency %)		Large genomic rearrangements		References
			BRCA1	BRCA2	BRCA1		BRCA2		BRCA1	BRCA2	BRCA1	BRCA2	
					Exon	Mutation	Exon	Mutation					
Brazil	54	a) Young female patient with BC diagnosed at < 35 year of age	6	4	5	c.181T>G	11	c.2808_2811delACAA	c.5266dupC (3.7%)	ND	NS	NS	Carraro et al. [13]
					7	c.560+2T>A	11	c.2494C>T					
		b) Women with a family history of BC			11	c.2405_2406delTG	11	c.4968 ins GT					
					11	c.3331_3334delCAAG	11	c.5190T>A					
					20	c.5266dupC							
					20	c.5251C>T							
Mexico	32	Early-onset BC patients (≤ 35 years) reporting no first or second-degree relatives with BC or OC	1	1	11	3587delT	11	c.519+5_519 + 8delGTAA	ND	ND	NS	NS	Ruíz-Flores et al. [48]
Mexico	22	Early-onset BC patients (≤ 35 years) with a family history of BC	1	1	11	3587delT	11	2664InsA	ND	ND	NS	NS	Calderón-Garcidueñas et al. [49]
Mexico	810	Early-onset BC patients (≤ 40 years) reporting no first or second-degree relatives with BC or OC	6	2	9_12	c.548-?_4185+?del	10	c.1796-1800delTTTAT	c.548-?_4185+?del (0.25%)	ND		NS	Torres-Mejia et al. [50]
					11	c.2296-2297delAG	11	c.4111C>T					
					11	c.2433delC							
					11	c.3598C>T							
					11	c.4327C>T							
					18	c.5123C>A							

*ND* not detected, *NS* not studied, *BC* breast cancer

**Table 2:** Cohort characteristics and pathogenic BRAC1 and BRAC2 mutation in unselected breast cancer cases in Central and South American populations

Country	Cohort size	Inclusion criteria	Number of mutation detected		Pathogenic mutation in BC patients				Recurrent mutation (frequency %)		Large genomic rearrangements (frequency %)		References
			BRCA1	BRCA2	BRCA1		BRCA2		BRCA1	BRCA2	BRCA1	BRCA2	
					Exon	Mutation	Exon	Mutation					
Brazil	402	Unselected, but all of the patients postive for a BRCA mutation had a family history of BC	2	2	11	c.3228_3229delAG	11	c.5946delT	c.5266dupC (1.2%)	c.6405_6409delCTTAA (0.5%)	NS	NS	Gomes et al. [37]
					20	c.5266dupC	11	c.640_6409delCTTAA					
Colombia^b^	766	Unselected for family history	2	1	11	c.3331_3334delCAAG c.5123C>A	11	c.2808_2811delACAA	c.3331_3334de lCAAG (1.6%) c.5123C>A (1.3%)	c.2808_2811delACAA (1.3%)	NS	NS	Torres et al. [44]
Colombia^c^	96	Unselected for family history	3	2	11	c.3331_3334de lCAAG	11	c.6024dupG	c.3331_3334de lCAAG (11.4)	ND	NS	NS	Rodríguez et al. [42]
					11	c.1674_1674de lA	11	c.6024dupG					
					18	c.5123C>A							
Colombia	244	Unselected for family history	2	1	11	c.3331_3334de lCAAG	11	c.5616_5620de lAGTAA	ND	ND	NS	NS	Hernández et al. [43]
					18	c.5123C>A							
Mexico	188	Unselected for family history	14	6	2	70insAG	10	1803insA	ND	ND	ex9-12del (6.9%)	ND	Villarreal-garza et al. [51]
					2	c.68_69de lAG	11	2900de lCT			ex8-9dup (1.1%)		
					5	c.211A>G	11	C.6024dupG			ex18-19del (1.1%)		
					5	c.212+1G>A	11	6244de lG			ex8-10del		
					11	c.798_799de lTT	11	c.6486_6489de lACAA					
					11	803de lA	25	c.9463_9467de I5in8					
					11	c.815_824dupAGCCATGTGG							
					11	c.2806_2809de lGATA							
					11	c.3759_3760de lTGAG							
					11	c.3858_3861de lTGAG							
					11	c.4065_4068de lTCAA							
					13	c.4327C>T							
					18	c.5095C>T							
					18	c.5123C>A							
Mexico	810	Unselected [85.3% with sporadic BC and 67.7% with early-onset BC (≤ 50 years of age)]	8	11	9_12	c.548?_4185?de l	10	c.1796-1800de lTTTAT	c.548?_4185?de I(1%)	c.1796-1800de lTTTAT(0.37%)	NS	NS	Torres-Mejia et al. [50]
					11	c.1016-1017insA	11	c.2808_2811de lACAA	c.2433de IC(0.25%)	c.4111C>T			
					11	c.2071-2071de lA	11	2971de I5	c.4327C>T (0.25%)				
					11	c.2296-2297de lAG	11	c.3264_3265insT	c.5123C>A (0.5%)				
					11	c.2433de lC	11	c.4111C>T					
					11	c.3598C>T	11	4321insAA					
					13	c.4327C>T	11	4534de lAT					
					18	c.5123C>A	11	c.5542de lA					
							11						
							11						
							11						
Peru^a^	266	Unselected for family history	4	1	2	c.68_69de lAG	11	c.2808_2811de lACAA	c.68_69de lAG (2.6%)	c.2808_2811de lACAA (0.75%)	NS	NS	Abugattas et al. [52]
					11	c.815_824dupAGCCATGTGG			c.1961_1962de lA (0.75%)				
					11	c.1961_1962de lA							
					11	c.3759_3706de lTA							
Peru^a^	124	Unselected, but 39.39% of patients had a positive family history of BC and/or OC	5	2	2 11 11 17	c211A>G c.4041_4042del c.4065_4068de lTCAA c.5074+1G>T c.5091_5092del	11 16	c2455C>T c.7673_7674de l	ND	ND	Del exon 18–19 Del exon 8–13	ND	González-Rivera et al. [53]

*ND* not detected, *NS* not studied, *BC* breast cancer

^a^A panel of BRCA1 and BRCA2 mutation wa used

^b^Only mutations previously described by Torres et al. [41] were studied

^c^A panel of 96 Hispanic BRCA mutation was used

Additional file 1: Table S1; Tables 1 and 2 show the cohort size, inclusion criteria, and *BRCA* pathogenic point mutations, LGR(s) and recurrent mutations detected in cohorts A, B and C, respectively. Additional file 1: Table S1 show that in hereditary BC, 118 different BRCA point mutations were detected in 9 countries (68 in *BRCA1* and 50 in *BRCA2*). Recurrent mutations were detected in Argentina, Chile, Brazil, Colombia and Costa Rica. Table 1 shows that in early-onset BC, 21 different *BRCA* mutations were detected in Brazil and Mexico (13 in *BRCA1* and 8 in *BRCA2*). The c.5266dupC and c.548-?_4185+?del mutations were recurrent in Brazil and Mexico, respectively. Table 2 shows that in cohorts unselected for family history, 51 different *BRCA* mutations (29 in *BRCA1* and 22 in *BRCA2*) were detected in Brazil, Colombia, Mexico and Peru. Large genomic rearrangements were reported in Argentina, Brazil, Chile, Mexico and Peru.

When the results were analyzed separately for each country, we found that 57 different *BRCA* mutations were detected in Argentina (32 in *BRCA1* and 25 in *BRCA2*), all in hereditary BC cohorts (n = 40), including 4 recurrent mutations (2 in *BRCA1* and 2 in *BRCA2*). Four LGRs were reported in *BRCA1* but none in *BRCA2* [29].

In Brazil, 6 studies that collectively screened 1151 individuals with hereditary BC reported 34 different *BRCA* mutations (24 in *BRCA1* and 10 in *BRCA2*) [30–35], including 7 recurrent mutations (5 in *BRCA1* and 2 in *BRCA2*) (Additional file 1: Table S1). In cohort B, a study by Carraro et al. [36] (n = 54) detected another 5 mutations (2 in *BRCA1* and 3 in *BRCA2*), including the recurrent mutation c.5266dupC (3.7%), which was also a recurrent mutation in hereditary BC (Additional file 1: Table S1). Another 3 mutations not seen in cohorts A or B were detected in cohort C (n = 402) (1 in *BRCA1* and 2 in *BRCA2*), including the recurrent mutation c.6405_6409delCTTAA (0.5%) [37]. Therefore, 42 different pathogenic point mutations in *BRCA* were described in the cohorts A, B and C in Brazil. All patients positive for *BRCA* mutations had a family history of BC (Additional file 1: Table S1; Tables 1, 2). Four different LGRs (3 in *BRCA1* and 1 in *BRCA2*) were also reported, all in hereditary BC, one of which was recurrent (Additional file 1: Table S1).

In Chile, 19 *BRCA* mutations were reported (9 in *BRCA1* and 10 in *BRCA2*), all in hereditary BC. Of these, 9 were recurrent (4 in *BRCA1* and 5 in *BRCA2*) (Additional file 1: Table S1) [38, 39]. Furthermore, 2 LGRs were detected in cohort A [40]. No *BRCA* mutations were reported in cohorts B or C.

The only study on patients with hereditary BC in Colombia (n = 53) described 6 *BRCA* mutations (2 in *BRCA1* and 4 in *BRCA2*), 2 of which were recurrent in *BRCA1* (c.3331_3334delCAAG and c.5123C>A) and one of which was recurrent in *BRCA2* (c.2808_2811delACAA) (Additional file 1: Table S1) [41]. Another 3 studies that collectively screened 1106 patients unselected for family history described another 4 mutations (1 in *BRCA1* and 3 in *BRCA2*) [42–44]. Table 3 shows the mutations that were reported in more than one cohort. No LGR studies were performed in Colombia. Therefore, in the Colombian population, 10 different pathogenic point mutations in *BRCA* were detected, 3 of which were recurrent (Additional file 1: Table S1 and Table 2), and no LGR studies were available.

**Table 3:** Mutations present in more than one cohort

Country	Mutation	Exon	Hereditary BC	Early-onset BC	Unselected BC
BRCA1					
Brazil	c.5266dupC	20	✔^a^	✔^a^	✔^a^
Brazil	c.560+2T>A	7	✔	✔	
Brazil	c.3331_3334delCAAG	11	✔	✔	
Brazil	c.5251C>T	20	✔	✔	
Colombia	c.3331_3334delCAAG	11	✔^a^		✔^a^
Colombia	c.5123C>A	18	✔^a^		✔^a^
Mexico	c.548?_4185?del	9_12		✔	✔
Mexico	c.4065_4068delTCAA	11	✔		✔
Mexico	c.2296-2297delAG	11		✔	✔
Mexico	c.2433delC	11	✔	✔	✔
Mexico	c.3598C>T	11		✔	✔
Mexico	c.4327C>T	13		✔	✔
Mexico	c.5123C>A	18	✔	✔	✔
Mexico	c.211 A>G	5	✔		✔
Mexico	c.3759_3760delTA	11	✔		✔
BRCA2					
Brazil	c.2808_2811delACAA	11	✔	✔	
Colombia	c.2808_2811delACAA	11	✔^a^		✔^a^
Mexico	c.2808_2811delACAA	11	✔		✔
Mexico	c.1796-1800delTTTAT	10		✔	✔^a^
Mexico	c.4111C>T	11		✔	✔

*BC* breast cancer

✔ = Mutation present

^a^Recurrent mutation

Only one study reported on *BRCA* mutations in Costa Rica. This study described 4 mutations (1 in *BRCA1* and 3 in *BRCA2*) in a heredity BC cohort (n = 111), including the recurrent mutation c.5303_5304delTT (1.8%) [45].

In Mexico, 17 different *BRCA* mutations were reported in hereditary BC (10 in *BRCA1* and 7 in *BRCA2*). Three LGRs were also described. The authors did not report recurrent mutations [46, 47]. In cohort B, 11 mutations were described (7 in *BRCA1* and 4 in *BRCA2*) [48–50]. Of these, 4 mutations in *BRCA1* (c.548-?_4185+?del, c.2296-2297delAG, c.3598C>T and c.4327C>T) and 3 in *BRCA2* (c.519+5_519+8delGTAA, c.1796-1800delTTTAT and c.4111C>T) were present in women with early-onset BC and no family history of the disease [48, 50]. In the Mexican patients unselected for family history, 36 different *BRCA* mutations were described (20 in *BRCA1* and 16 in *BRCA2*) [50, 51]. Of these, 12 were also present in cohorts A or B (Table 3). In cohort C, 6 point mutations were recurrent (4 in *BRCA1* and 2 in *BRCA2*), including c.548-?_4185+?del, which was also a recurrent mutation in early-onset BC patients with no family history of the disease. In cohort C, 3 recurrent LGRs were reported. The LGR exon 9-12del had a frequency of 6.9%, making it one of the most frequent *BRCA* mutations described in the Mexican population.

Three studies were available for Peru. Two studies with cohorts unselected for family history of BC reported 12 different mutations (9 in *BRCA1* and 3 in *BRCA2*). The mutations c68_69delAG, c.1961_1962delA and c.2808_2811delACAA were recurrent, and 2 LGRs were also detected (Table 2) [52, 53]. The third publication tested for LGRs in 16 hereditary BC patients but did not test for pathogenic point mutations. The authors detected only one LGR, in *BRCA1* (exon 7 amplification) [54].

In Uruguay, only one study described *BRCA* mutations, in a cohort of 53 patients with heredity BC. Seven mutations were detected (2 in *BRCA1* and 5 in *BRCA2*), and no LGR testing was performed [55].

In Venezuela, only one study reported *BRCA* mutations, again in patients with hereditary BC (n = 51). The authors described 6 different mutations (3 in *BRCA1* and 3 in *BRCA2*). No recurrent mutations were reported, and no LGR testing was performed [56].

Table 4 shows *BRCA1/2* mutations common in more than one Central or South American country, including a total of 21 mutations (14 in *BRCA1* and 7 in *BRCA2*). The most common mutations were found in exons 2, 5, 11, 13, 18 and 20 in *BRCA1* and in exons 3 and 11 in *BRCA2*. Seven mutations were present in 3 or more countries: c.68_69delAG, c.211A>G, c.3331_3334delCAAG and c.5123C>G in *BRCA1* and c.145G>T, c.2808_2811delACAA and c.5946delT in *BRCA2*. The c.68_69delAG mutation, also known as 185delAG (*BRCA1* exon 2), was described in Argentina, Brazil, Chile, Mexico and Peru and was reported as a recurrent mutation in Brazil (0.3%), Chile (0.6%) and Peru (2.6%). The mutation c.211A>G (*BRCA1* exon 5) was detected in Argentina, Brazil, Mexico and Peru and was reported as a recurrent mutation in hereditary BC in Argentina (1.17%). The c.3331_3334delCAAG was present in BC patients from Brazil, Chile and Colombia and was a recurrent mutation in Chile (0.9%) and Colombia (9.4%). The mutation c.5123C>A (*BRCA1* exon 18) was detected in Argentina (cohort A), Brazil (Cohort A), Colombia (cohort A and C) and Mexico (cohort A, B and C) and was a recurrent mutation in Colombia (5.7%) and Mexico (0.5%). In *BRCA2*, 6 mutations in exon 11 (c.2808_2811delACAA, c.3264dupT, c.4740_4741insTG, c.535dupA, c.5946delT and c.6024dupG) and one in exon 3 (c.145G>T) were detected in more than one country; c.2808_2811delACAA was a recurrent mutation in Argentina (0.64%), Colombia (3.8%) and Peru (0.75%), and c.145G>T was a recurrent mutation in Chile (2.6%).

**Table 4:** Common BRCA ½ mutation found in multiple Central and South American countries

Mutation in BRCA 1						Frequency of recurrent mutation (%)		
Exon	Mutation	Country	Hereditary	Early-onset BC	Unselected BC	Hereditary BC	Early-onset BC	Unselected BC
2	c.68_69delAG	Argentina	✔					
		Brazil	✔			0.33%		
		Chile	✔			0.6%		
		Mexico			✔			
		Peru			✔			2.6%
5	c.181T>G	Argentina	✔			0.64%		
		Brazil		✔				
		Chile	✔					
5	c.211A>G	Argentina	✔			1.17%		
		Brazil	✔					
		Mexico	✔		✔			
		Peru			✔			
11	c. 798_799delTT	Argentina	✔					
		Mexico			✔			
11	c.815_824dupAGCCATGTGG	Mexico			✔			
		Peru			✔			
11	c.2568T>G	Argentina	✔					
		Uruguay	✔					
11	c.3228_3229delAG	Argentina	✔					
		Brazil			✔			
11	c. 3331_3334delCAAG	Brazil	✔					
		Chile	✔			0.9%		
		Colombia	✔		✔	9.4%		1.6%/11.4%^a^
11	c. 3858_3861delTGAG	Mexico	✔		✔			
		Peru			✔			
11	c. 3858_3861delTGAG	Chile	✔					
		Mexico			✔			
11	c. 4065_4068delTCAA	Mexico			✔			
		Peru			✔			
13	c.4327>T	Argentina	✔					
		Mexico		✔	✔			0.25%
18	c. 5123C>A	Argentina	✔					
		Brazil	✔					
		Colombia	✔		✔	5.7%		1.3%
		Mexico	✔	✔	✔			0.5%
20	c.5266up C	Argentina	✔					
		Brazil	✔	✔	✔	2.5%/0.65%/5%^a^	3.7%	1.2%
Mutation in BRCA 2								
3	c.145G>T	Argentina	✔					
		Chile	✔			3.7%		
		Mexico	✔					
11	c.2808_2811 delACAA	Argentina	✔			0.64%		
		Brazil	✔	✔				
		Colombia	✔		✔	3.8%		1.3%
		Mexico	✔		✔			
		Peru			✔			0.75%
		Venezuela	✔					
11	c.3264d up T	Argentina	✔					
		Mexico	✔					
11	c.4740_4741insTG	Argentina	✔					
		Chile	✔			0.6%		
11	c.5351dup A	Argentina	✔					
		Uruguay	✔					
11	c. 5946delT	Argentina	✔					
		Brazil			✔			
		Chile	✔					
		Costa Rica	✔					
11	c.6024dup G	Argentina	✔					
		Colombia			✔			
		Mexico			✔			

*BC* breast cancer

✔ = Mutation present

^a^Values obtained in different publication

### Other BC susceptibility mutations in Central and South American countries

There is a consensus that BC risk is attributable to susceptibility alleles in many different genes. In patients negative for *BRCA1/2* mutations, inherited variations in other genes explain up to 20% of familial BC [8]. However, 51% of breast cancer families do not show mutations in *BRCA1/2* or other known susceptibility genes and are therefore classified as BRCAX families. These families may carry a mutation in a moderate-penetrance BC gene yet to be identified. Alternatively, a truly polygenic model may underlie these cases, with susceptibility conferred by the collective actions of several low-penetrance loci [57–60]. We carried out a literature review of reports on pathogenic mutations or variants in other susceptibility genes in Central and South American countries and found 19 publications between January 2002 and February 2017 in 5 Central or South American countries: Brazil, Chile, Ecuador, Mexico and Peru (Fig. 1). Pathogenic mutations or variants that increase BC risk were reported in the following genes or genomic regions: *ATM, BARD1, CHECK2, FGFR2, GSTM1, MAP3K1, MTHFR, PALB2, RAD51, TOX3, TP53, XRCC1* and 2q35.

*ATM* is frequently implicated in hereditary BC as a low-penetrance susceptibility gene. The *ATM* kinase has an essential role maintaining genomic integrity, as a key activator of cellular responses to DNA double-strand breaks [61]. In Chile and Mexico, association studies were performed to evaluate the relationship between common *ATM* variants and familial BC [62, 63]. The same variants were studied in both countries: IVS24-9delT and IVS38-8T>C. Both reports concluded that these variants are associated with increased risk of BC (Table 5). In Chile, the authors studied the variant 5557G>A, which was also found to increase BC risk [62].

**Table 5:** Mutations or variations in other breast cancer susceptibility genes in Central and South American populations

Country	Cohort size	Selection criteria	BC susceptibility gene		References
			Gene	Mutation or variant	
Brazil	874	a) Family history of BC b) Unselected for family history	TP53	c.1010G>A (pathogenic mutation) Frequency: 8.23%	Giacomazzi et al. [85]
Brazil	120	a) BC diagnosed at ≤ 45 years of age (no family history of BC) b) BC diagnosed at ≤ 45 years of age; at least 1 close blood relative with breast/ovarian/fallopian tube/primary peritoneal cancer diagnosed at any age c) BC diagnosed at ≤ 50 years of age; at least 1 blood relative with breast/ovarian/fallopian tube/primary peritoneal cancer diagnosed at ≤ 50 years d) BC diagnosed at > 50 of age; at least 1 blood relative with breast/ovarian/fallopian tube/primary peritoneal cancer diagnosed at any age e) At least 2 relatives with primary BC diagnosed at < 50 years of age f) BC with a history of ovarian/fallopian tube/primary peritoneal cancer diagnosed at any age g) Ethnicity associated with a higher mutation frequency (e.g., Ashkenazi Jewish) h) Personal history of ovarian/fallopian tube/primary peritoneal cancer i) Personal history of male BC	TP53 CHEK2	c.1010G>A (pathogenic mutation) Frequency: 2.5% c.1100delC Frequency: 0.83%	Silva et al. [31]
Brazil	348	Female with BC diagnosed at < 45 years of age; no family history of the disease	TP53	c.1010G>A (pathogenic mutation) Frequency: 12%	Andrade et al. [78]
Brazil	100	Patient with BC; no family history of the disease	MTHFR	MTHFR c.677T (rs1801133) associated with increased BC risk	Zara-Lopes et al. [77]
Brazil	49	a) Women with family history of BC b) Women with no family history of BC	GSTM1	Null GSTM1 associated with increased BC risk	Possuelo et al. [73]
Chile	143	a) At least 2 first-degree relatives with BC and/or OC diagnosed at any age (46.1%) b) At least 2 first- or second-degree relatives with BC diagnosedat < 50 years of age (22.7%) c) At least 1 relative with BC diagnosed at < 30 of age years (11.3%) d) At least a relative with bilateral BC e) At least 3 first- or second-degree relatives with BC; at least 1 diagnosed at < 40 years of age (5.7%) f) 3 or more different cancers (female or male BC, OC, prostate, pancreatic or larynx in non-smoking individuals) (5.7%) g) At least 1 relative with male BC diagnosed at any age; at least 1 relative with female BC diagnosed at any age	RAD51	RAD51 135G>C associated with increased BC risk in BRCA1/2 negative women with a family history of BC and diagnosis at < 50 years of age	Jara et al. [81]
Chile	137	a) At least 2 relatives with BC b) At least 2 relatives with BC; at least 1 with diagnosis at < 40 years of age c) At least 2 relatives with BC; at least 1 relative with bilateral BC d) At least 3 relatives with BC e) At least 3 relatives with BC; at least 1 with diagnosis at < 40 years of age f) At least 3 relatives with BC; at least 1 male relative with BC g) At least 3 relatives with BC and/or OC h) Two family members with BC; at least one with both BC and OC i) At least 1 relative with BC diagnosed at < 31 years of age; male BC	ATM	IVS24-9delT IVS38-5557G>A all associated with increased BC risk	González-Hormazabal et al. [67]
Chile	322	a) At least 3 relatives with BC and/or OC b) 2 relatives with BC and/or OC c) At least 1 relative with BC diagnosed at ≤ 35 years of age d) At least 1 relative with BC diagnosed at ≤ 36–50 years of age	BARD1	BARD1 Cys557Ser associated with increased BC risk	González-Hormazabal et al. [66]
Chile	351	a) At least 3 relatives with BC and/or OC b) 2 relatives with BC and/or OC c) At least 1 relative with BC diagnosed at ≤ 35 years of age d) At least 1 relative with BC diagnosed at ≤ 36–50 years of age	FGFR2 MAP3K1	rs2981582, rs2420946 and rs1219648 All associated with increased BC risk rs889312 associated with increased BC risk	Jara et al. [92]
Chile	347	a) At least 3 relatives with BC and/or OC b) 2 relatives with BC and/or OC c) At least 1 relative with BC diagnosed at ≤ 35 years of age d) At least 1 relative with BC diagnosed at ≤ 36–50 years of age	TOX3 2q35	rs3803662 associated with increased BC risk rs13387042 associated with increased BC risk	Elematore et al. [94]
Chile	436	a) At least 3 relatives with BC and/or OC b) 2 relatives with BC and/or OC c) At least 1 relative with BC diagnosed at ≤ 35 years of age d) At least 1 relative with BC diagnosed at ≤ 36–50 years of age	PALB2	rs152451 and rs45551636 associated with increased BC risk in cases with strong family history of BC	Leyton et al. [95]
Chile	196	BC patients belonging to a high-risk family	CHEK2 1100delC	Not detected	González-Hormazabal et al. [67]
Ecuador	114	Unselected for family history of cancer	MTHFR	MTHFR c.677T (rs1801133) associated with increased BC risk	López-Cortes et al. [78]
Mexico	397	Unselected for family history of cancer	XRCC1	Arg399Gln associated with increased BC risk	Macías-Gómez et al. [91]
Mexico	559	Unselected for family history of cancer	GSTM1	Null GSTM1 associated with increased BC risk	Soto-Quintana et al. [69]
Mexico	243	Unselected for family history of cancer	GSTM1	Null GSTM1 associated with increased BC risk	Jaramillo- Rangel et al. [72]
Mexico	94	Familial and/or early-onset BC	ATM	IVS24-9delT IVS38-5557G>A all associated with increased BC risk	Calderón-Zúñiga et al. [63]
Mexico	687	Unselected for family history of cancer	FGR2	rs2981582 associated with increased BC risk	Murillo-Zamora et al. [93]
Peru	105	a) Triple-negative BC b) Unselected for family history of cancer or age at diagnosis (but 39.39% had a family history of breast or ovarian cancer)	BARD1 RAD51D	c.334C>T (pathogenic) Frequency: 0.95% c.694C>T (pathogenic) Frequency: 0.95%	González-Rivera et al. [53]

Germline and somatic mutations in the *BARD1* gene are reportedly associated with susceptibility to a subset of breast and ovarian cancers [64]. *BARD1* participates in important cellular processes such as DNA repair, RNA processing, transcription, cell cycle regulation and apoptosis [65]. Studies on *BARD1* were performed in Chile and Peru (Table 5) [53, 66]. Gonzalez-Hormazabal et al. [66] reported that in Chilean women negative for BRCA1/2 mutations, *BARD1* Cys557Ser was associated with increased risk of BC. In Peru, one pathogenic mutation (c.334C>T) was reported in one of the triple-negative BC patients studied (0.95%).

*CHEK2* is a gene involved in DNA damage and replication checkpoint responses and has been suggested as a BC susceptibility gene. The *CHEK2* 1100delC variant, which is associated with increased BC susceptibility among familial BC cases not attributable to mutations in *BRCA1/2* [67], was studied in Brazilian (n = 120) [31] and Chilean (n = 196) patients with hereditary BC [67]. Only one of the Brazilian patients carried this mutation (0.83%), and it was not present is any of the Chilean cases (n = 196). Therefore, this variant is not a common mutation in these two populations (Table 5).

Glutathione S-transferases (*GSTs*) play an important role in carcinogen detoxification and metabolism of various bioactive compounds [68]. The GST family is composed of six classes of isoenzymes, including *GSTM1* [69]. The *GSTM1* gene is polymorphic in humans and has three known alleles: GSTM1*A, GSTM1*B and *GSTM1O* (null), which is the most common variant. The null variant results in undetectable expression of the gene product [70], leading to excessive accumulation of reactive oxygen species and consequently higher susceptibility to carcinogenic events due to DNA damage [71]. Three studies in Mexican and Brazilian populations evaluated the association between the null genotype and BC risk. Two reports concluded that *GSTM1O* is associated with BC risk in patients from northeastern Mexico [72] and Guadalajara [69]. In Brazil, a study by Possuelo et al. [73] also reported an association between the null *GSTM1* genotype and BC risk.

The *MTHFR* enzyme, encoded by the *MTHFR* gene, is responsible for catalyzing the irreversible conversion of 5,-0-methylenetetrahydrofolate to 5-methylenetetrahydrofolate. The latter molecule is involved in DNA methylation, an important mechanism in regulation of gene expression. Alterations in DNA methylation due to *MTHFR* polymorphisms may be associated with the development of cancer [74–76]. Association studies on *MTHR* C677T polymorphisms and BC risk were performed in Brazil [77] and Ecuador [78] (Table 5). In both reports, the authors found a significant association between this SNP and BC risk.

*RAD51* is a gene that plays a key role in repairing DNA double-strand breaks through homologous DNA recombination, forming complexes with other proteins involved in DNA repair such as *BRCA2* [79, 80]. Variants or pathogenic mutations in this gene were studied in Chile [81] and Peru [53]. In Chile, no mutations were detected in the exon or splice-boundaries regions of the *RAD51* gene. The same study also evaluated the *RAD51* 5′UTR variant 135 G>C, which is associated with an increased risk of familial BC in *BRCA1/2*-negative women and early-onset BC (age < 50 years at diagnosis). In Peru, the pathogenic mutation c.694C>T was detected in triple-negative BC patients (n = 105), with a frequency of 0.95% (Table 5).

Mutations in the *TP53* tumor suppressor gene also play a significant role in cancer risk, as impaired p53 function may contribute to the multistep process of carcinogenesis [82]. The p53 protein is important in cell-cycle regulation and maintenance of genome stability. The most notable property of p53 is its action as a transcription factor [83]. We found three articles that studied variations in *TP53*, all in Brazilian populations [31, 84, 85]. These articles studied the c.1010G>A (p.R337H) mutation, which occurs at a high frequency in southern and southeastern Brazil [86–90]. Silva et al. [31] reported a frequency of 2.5% for this variant and suggested that all BRCA-negative female BC patients with clinical criteria for hereditary breast-ovarian cancer should be tested for the c.1010G>A variant. Giacomazzi et al. [84] reported that the prevalence of p.R337H was higher in women diagnosed with BC at or before 45 years of age (12.1%) than in those diagnosed at 55 or older (5.1%). An article by Andrade et al. [85] suggested that screening for the germline *TP53* p.R337H mutation should be recommended for young females with no family history of cancers associated with Li-Fraumeni syndrome. The three authors agree that inheritance of the c.1010G>A variant may significantly contribute to the high incidence of BC in Brazil.

The *XRCC1* gene encodes a protein involved in DNA base excision repair. Therefore, mutations or polymorphisms in this gene may be involved in the genetic etiology of BC. The only study on the association between the *XRCC1* gene and BC risk was performed in a Mexican population [91]. Macias-Gomez et al. [91] studied Arg1945Trip and Ag399Gln, reporting a significant association between BC risk and the 399Gln polymorphism but no significant association with the Arg194Trip polymorphism.

Variations in the *FGFR2* gene were studied in Chile [92] and Mexico [93]. The genes or genomic regions in *MAP3* *K, TOX3, PALB2*, *2q35* and *8q24* were studied only in Chile (Table 5) [92, 94, 95].

Fibroblast Growth Factor Receptor 2 (*FGFR2*) and mitogen-activated protein kinase-kinase-kinase 1 (*MAP3K1*) have been proposed as low-penetrance BC susceptibility genes [57]. A study by Jara et al. [92] used a case–control design to evaluate the association of BC with the *FGFR2* SNPs rs2981582, rs2420946 and rs121648 and the *MAP3K1* SNP rs889312 in BRCA1/2-negative Chilean BC cases. All of the SNPs studied were significantly associated with increased BC risk in familial BC and non-familial early-onset BC, in a dose-dependent manner. In Mexico, a study by Murillo-Zamora et al. [93] reported that rs2981582 was associated with BC risk (p = 0.007) (Table 5).

In the *TOX3/LOG643714* (also known as *TNRC9*) locus, several SNPs associated with BC risk were identified. Among these, rs380362 is the most strongly correlated with disease [57]. The SNPs rs13387042 (2q35) and rs13281615 (8q24), located in non-coding regions, were also associated with BC risk [57, 60]. In a Chilean population, Elematore et al. [94] evaluated the association between rs380362 (*TOX3*), rs13387042 (2q35) and rs13281615 (8q24) and BC risk in 344 *BRCA1/2*-negative BC cases and 801 controls. Two SNPs, rs380362 and rs13387042, were significantly associated with increased BC risk in familial BC and non-familial early-onset BC. The risk of BC increased in a dose-dependent manner with the number of risk alleles (p-trend < 0.0001 and 0.0091, respectively). Other studies reported an additive effect of the rs380362 and 2q35 rs1387042 alleles on BC risk. There was no association between rs13281615 (8q24) and BC risk (Table 5).

The PALB2 (partner and localizer of BRCA2) protein interacts with BRCA2, stabilizing the intracellular accumulation of the BRCA2 protein at sites of DNA damage [96]. PALB2 is also recruited by BRCA1 in response to DNA damage and serves as a linker between BRCA1 and BRCA2 and is necessary for BRCA2-mediated homologous-recombination repair [97, 98]. Thus, *BRCA1*, *BRCA2* and *PALB2* are key BC susceptibility genes that work together in the same DNA damage response pathway [99, 100]. Leyton et al. [95] studied 100 *BRCA1/2*-negative Chilean cases with familial BC, identifying 3 *PALB2* variants. Using a case–control design, the authors evaluated the association of the identified variants with BC risk. Two of the variants, *PALB2* c.1676A>G(rs152451A>G) and c.2993C>T (rs45551636C>T), were significantly associated with increased BC risk only in cases with a strong family history of BC (Table 5).

### The relationship of *BRCA1/2* mutations and other BC susceptibility variants to the demographic composition of Central and South American countries

Genetic factors play an important role in the development of BC. The most widely-accepted model of BC oncogenesis, known as the polygenic model, attributes BC susceptibility to a small number ethnicity-specific mutations in high-penetrance genes (*BRCA1*, *BRCA2* and *TP53*) and a much larger number of variants in moderate- or low-penetrance genes [7, 101], as well as interactions among these genetic variants and exposure to environmental factors [102]. Both *BRCA1* and *BRCA2* confer susceptibility to breast and ovarian cancer. About 5–7% of all BC diagnosed are associated with germline mutations in *BRCA1* and *BRCA2* [8, 15], and an even larger proportion of familial BC cases are associated with *BRCA1* and *BRCA2* variations; collectively, germline mutations in the two major susceptibility genes *BRCA1* and *BRCA2* account for ~ 20% of familial BC cases [8, 103]. The spectrum of mutations in *BRCA1* and *BRCA2* genes and other susceptibility alleles varies considerably by ethnic group and geographic region.

South America has a complex demographic history shaped by multiple migration and admixture events in pre- and post-colonial times [104], including settlement by Native Americans, European colonization and the African slave trade [104]. Moreover, the continental ancestry of the admixed populations in South America is not homogenous. For example, the Argentine population is a mixture of European (0.673), Native American (0.277), West African (0.036) and East Asian (0.014) components, while the proportions in the Peruvian population are European (0.26), Native American (0.683), West African (0.032) and East Asian (0.025) [104]. Uruguay is unique among South American countries in that it has almost no communities of Native American or African descent [105]. Therefore, South American countries should not be analyzed as a monolithic group without regard for specific regional genetic ancestry, as the ethnic differences between South American populations suggests that medically-relevant genetic variations may differ according to population and region.

Mexico and Costa Rica were the only Central American populations with data on BRCA mutations. Central America was included in this review as it was also colonized by Spaniards. The Costa Rica population is a mixture of European (0.61), Native American (0.31) and African (0.06) components, with variations by region [106]. For example, a recent study on the genetic and population substructure in Guanacaste, Costa Rica, which is heavily admixed, reported a mixture of predominantly European (0.425), Native American (0.383) and African (0.152) ancestry, although the authors could not exclude an Asian component (0.04) [107].

The Mexican population also harbors great ethnic diversity [108] as confirmed by numerous studies on the admixture in Mexico. Amerindian ancestry is the largest component (0.51–0.56) in the general population, followed by European (0.40–0.45), while the African component is small (0.02–0.05). When analyzed by region, however, there is significant variation. For example, European is the largest component in the north (at 0.5 in Chihuahua, 0.62 in Sonora and 0.55 in Nueva Leon) [105].

An overview of the literature indicates a marked Amerindian influence in Mexican and Peruvian populations, while European ancestry is more prevalent in Costa Rica, Argentina and Uruguay. The proportions of European, Amerindian and African components are roughly equal in Venezuela. In Colombia and Brazil, there is significant interpopulation variability. The ethnic distribution in Brazil follows a geographical pattern, with the European influence more prevalent in the southeast and south, African in northeast and Amerindian in the north. In Chile, the Amerindian and European components are 0.6 and 0.4, respectively [105].

### Genetic testing for breast cancer

Genetic testing for *BRCA1* and *BRCA2* mutations may provide significant public health benefits for cancer patients and high-risk individuals, who could be offered targeted treatment and prevention strategies [109]. The feasibility of providing widespread genetic screening for *BRCA1/2* mutations in Central and South America depends on knowledge of mutations present in these regions, given the varied ethnic composition of the populations. To develop a test that might be useful throughout the region and therefore sufficiently cost-effective, it is first necessary to determine which *BRCA1/2* mutations are common in multiple countries. Public insurance coverage for genetic testing is also crucial. Finally, it is important to identify pathogenic mutations or variants in other moderate- or low-penetrance susceptibility genes that increase BC risk, as the use of panel testing is growing more common.

## Conclusions

The BRCA1/2 gene mutation spectrum varies widely throughout different Central and South American populations, likely due to the patterns of ethnic diversity in these countries. These complex ethnic patterns are associated with various migration and settlement events. Even populations within a given country are not necessarily homogeneous, and each subgroup may have a distinct ethnic composition and genetic structure. Because the same genetic composition cannot be extrapolated across diverse sub-populations, genetic screening tests for breast cancer in these regions should not be based on a single genetic test with a defined gene variant panel to detect mutational events. This guideline is even more categorical for screening approaches designed to test more than one population in Central and or South American countries.

A significant percentage of high-risk families with hereditary breast cancer are negative for mutations in BRCA1/2 genes. The genetic etiology of BC in these subjects may be attributable to variations in other moderate- or low-penetrance susceptibility alleles and/or variations in specific chromosomal regions. Data on variants in these genes and/or chromosomal regions in Central and South American populations are even scarcer than studies involving high-penetrance alleles. Given the importance of these variants in the etiology of hereditary BC, elucidating the distribution of these mutations and variations is crucial for advancing population studies and screening approaches in high-risk families with a hereditary breast cancer profile.

Appropriate inclusion criteria are also of vital importance when conducting these studies, given the considerable variability observed in the reported studies.

## Additional file

**Additional file 1: Table S1.** Cohort characteristics and pathogenic BRCA1 and BRCA2 mutations in hereditary breast cancer in Central and South American populations.

## Abbreviations

BRCA1: breast cancer type 1 susceptibility protein
BRCA2: breast cancer type 2 susceptibility protein
LGRs: large genomic rearrangements
ATM: ataxia telangiectasia mutaded gene
BARD1: BRCA1 associated ring domain 1
CHEK2: Checkpoint kinase 2
GSTs: glutathione* S*-transferases
MTHFR: methylenetetrahydrofolate reductase
RAD51: BRCA1/BRCA2-containing complex, subunit 5
TP53: phosphoprotein P53
XRCC1: X-ray repair cross-complementing protein 1
FGFR2: fibroblast growth factor receptor 2
MAP3K1: mitogen-activated protein kinase-kinase-kinase 1
TOX3/LOG643714: TOX high mobility group box family member 3
PALB2: partner and localizer of BRCA2
